# Cigarette Smoking Is Associated with a Lower Concentration of CD105^+^ Bone Marrow Progenitor Cells

**DOI:** 10.1155/2015/914935

**Published:** 2015-08-09

**Authors:** Shaul Beyth, Rami Mosheiff, Ori Safran, Anat Daskal, Meir Liebergall

**Affiliations:** Department of Orthopedic Surgery Department, Hadassah-Hebrew University Medical Center, 91120 Jerusalem, Israel

## Abstract

Cigarette smoking is associated with musculoskeletal degenerative disorders, delayed fracture healing, and nonunion. Bone marrow progenitor cells (BMPCs), known to express CD105, are important in local trophic and immunomodulatory activity and central to musculoskeletal healing/regeneration. We hypothesized that smoking is associated with lower levels of BMPC. Iliac bone marrow samples were collected from individuals aged 18–65 years during the first steps of pelvic surgery, under IRB approval with informed consent. Patients with active infectious or neoplastic disease, a history of cytotoxic or radiation therapy, primary or secondary metabolic bone disease, or bone marrow dysfunction were excluded. Separation process purity and the number of BMPCs recovered were assessed with FACS. BMPC populations in self-reported smokers and nonsmokers were compared using the two-tailed *t*-test. 13 smokers and 13 nonsmokers of comparable age and gender were included. The average concentration of BMPCs was 3.52 × 10^5^/mL ± 2.45 × 10^5^/mL for nonsmokers versus 1.31 × 10^5^/mL ± 1.61 × 10^5^/mL for smokers (*t* = 3.2,  *P* = 0.004). This suggests that cigarette smoking is linked to a significant decrease in the concentration of BMPCs, which may contribute to the reduced regenerative capacity of smokers, with implications for musculoskeletal maintenance and repair.

## 1. Introduction

Cigarette smoking is known for its deleterious effect on many systems and organs. In comparison with research on the relationship of smoking and other organ systems, relatively little research has been performed with the aim of studying the effects of smoking on the musculoskeletal system, although there is evidence of impaired bone healing [1–5]. Smoking has been implicated in the early degeneration of mesenchymal tissues [6–9], delayed healing of injured tissues [3, 5, 10], and high complication rates in reconstructive surgical procedures [11–14]. These effects may result from decreased oxygen delivery to the tissues [15], decreased collagen production [16], altered levels of specific metalloproteinase enzymes [17], lower levels of cytokines and growth factors crucial for tissue regeneration [18], and a reduction in gene expression of bone morphogenetic proteins- (BMP-) 2, 4, and 6 [19], among other effects. The BMPs play a key role in regulation of the inflammatory response, chondrogenic stage, and osteogenic stage during fracture healing [20].

Bone marrow progenitor cells (BMPCs), also known as multipotent mesenchymal stromal cells, are rare multipotent cells residing in all musculoskeletal tissue that serve as a reservoir for tissue regeneration. They have been defined as (1) being adherent to plastic in culture, (2) expressing the surface markers CD105, CD90, and CD73 but not CD45, CD34, or CD14, and (3) being capable of differentiating into osteoblasts, adipocytes, and chondroblasts in vitro [21]. Due to their capability for multipotent differentiation into specialized cells and support of hematopoiesis and their secretion of a wide range of bioactive molecules that play an important role in local trophic and immunomodulatory activity, BMPCs are central to healing and regeneration following their recruitment to the site of injury [22–24]. It is therefore important to note that a bone fracture, like any other musculoskeletal injury, should be considered a systemic event, resulting in a systemic response [25].

Since these cells are rare, it has been difficult to isolate them in quantities that are large enough to evaluate their concentration. We have developed a safe and effective method for rapid isolation of CD105^+^ BMPCs from bone marrow aspirate. In a previous study, we have shown that cells in this population are plastic adherent, express CD105 and CD90 but not CD45, CD34, or CD14, and are multipotent [26], meeting the great majority of criteria for multipotent mesenchymal stromal cells [21]. This method enables us to obtain large numbers of BMPCs for both research and clinical use from a relatively small sample of bone marrow aspirate.

In the current study, we aimed to assess the concentration of BMPCs in bone marrow aspirate from patients undergoing orthopedic procedures. We hypothesized that cigarette smoking is associated with a decreased concentration of BMPCs, a possible contributor to their lower musculoskeletal regenerative capacity.

## 2. Patients and Methods

### 2.1. Patients

As part of a larger IRB-approved clinical study, we prospectively collected and analyzed bone marrow samples from 26 individuals undergoing pelvic surgery involving the iliac bone. Participants completed an informed consent. Inclusion criteria for patients in the current study were a clinical indication for pelvic surgery, age of 18–65 years, and completion of an informed consent for intraoperative aspiration of small quantities of bone marrow at the beginning of surgery; exclusion criteria were active infectious or neoplastic disease, a history of cytotoxic or radiation therapy, primary or secondary metabolic bone disease, and bone marrow dysfunction.

For patients included in the study, demographic and hematologic data were prospectively recorded. Patients were asked whether they currently smoked cigarettes. Following data collection, participants were divided into two groups, those who currently smoked a minimum of 20 cigarettes per day (current smokers) and those who had never smoked (nonsmokers). Data for patients who had previously smoked but had stopped were not included in the current analysis. Clinicians, laboratory personnel, and nursing staff were blinded to smoking status during data collection and analysis.

### 2.2. Hematology

A complete blood count (CBC) was routinely obtained for each patient prior to the intervention. Red and white blood cells and platelets were counted to rule out generalized bone marrow suppression in participants and to ensure that the smoking and nonsmoking cohorts had comparable hematological profiles.

### 2.3. Bone Marrow Aspiration

Bone marrow was aspirated from the iliac crest as a first step in the osseous procedure to ensure samples were representative of the bone marrow and avoid dilution with peripheral blood or mobilization of cells from the marrow. Bone marrow samples (15–20 mL) were obtained by staged aspiration using an 11 G Jamshidi bone-marrow needle (Angiotech, Vancouver, BC, Canada) and heparin-washed 20 mL syringe. Needle position was changed after aspiration of each 2–3 mL of bone marrow to avoid aspiration of peripheral blood. Each sample was numbered to enable blinding. The numbered sample was immediately placed in a 50 mL sterile tube containing 10 mL of low-glucose (1 g/L) Dulbecco's Modified Eagle's Medium (Biological Industries, Beit HaEmek, Israel) and 1 mL (5000 IU) of heparin and transferred in a cold icebox to a GMP-compatible laboratory for processing.

### 2.4. BMPC Isolation and Concentration

Our method for BMPC isolation was described earlier [26]. This is a positive selection assay in which we target CD105-positive (CD105^+^) cells from the mononuclear fraction derived from freshly aspirated bone marrow. Briefly, the aspirated bone marrow sample was washed with PBS. The recovered cells were collected by centrifugation at 900 g and loaded onto Percoll density gradient solution (density 1.077–1.080 3 g/mL). Cell separation was accomplished by centrifugation at 1100 g (30 min at 20–25°C). The collected nucleated cells were then washed twice with PBS and taken for isolation by magnet activated cell sorting (MACS) using CD105-microbeads (Miltenyi Biotec, Bergisch Gladbach, Germany). Nucleated cells were then resuspended in MACS buffer (0.5% BSA, 2 mM EDTA in 1 × PBS at pH 7.2) at a concentration of 10^7^ cells per 80 mL MACS buffer. Cells were mixed with CD105 microbeads (20 mL microbead solution per 10^7^ nucleated cells) and incubated on a rotator at 4°C for 30 minutes in the dark. CD105^+^ cells were then selected by passing the nucleated cells through a separation column (Miltenyi Biotech) placed on a magnet, followed by elution using MACS buffer when the column was no longer on the magnet. Both fractions of nucleated cells were counted and then subjected to FACS analysis to ascertain their phenotype as CD105^+^ or CD105-negative (CD105^−^).

The advantage of this unique method is the short time required to isolate significant quantities of fresh, viable BMPCs from bone marrow and the ability to obtain a more direct count of the BMPC population in a fresh sample. Cells obtained in this method were shown to have the capability of differentiation and proliferation, as described previously by our group [26].

### 2.5. Statistical Analysis

Data for the smoking and nonsmoking groups were compared for demographic details, hematology parameters, and BMPC concentration. Differences in continuous outcomes were assessed using the two-tailed *t*-test, and differences between categorical outcomes were measured with the chi-square test.

## 3. Results

### 3.1. Patients

The average age of patients was 39 and 36 years for current smokers and nonsmokers, respectively. The two groups were comparable with regard to age and gender distribution (Table 1).

Average values of hematological parameters, including hemoglobin, red blood cells, mean corpuscular volume, white blood cell, and platelet counts were comparable for the two groups and within normal limits (Table 2). This is important, since the bone marrow serves as a reservoir for mesenchymal stem cells and bone marrow dysfunction, either overproduction of cells or hypocellularity, might indicate imbalance or acute changes, thereby confounding the results. Average monocyte concentration, which includes the BMPC population, was also comparable in the two groups (Table 2). A trend of lower thrombocyte count in current smokers did not reach statistical significance, but thrombocyte count may be found in the future to be related to the marrow's well-being.

### 3.2. Efficacy of BMPC Isolation and Concentration

**Figure 1:**
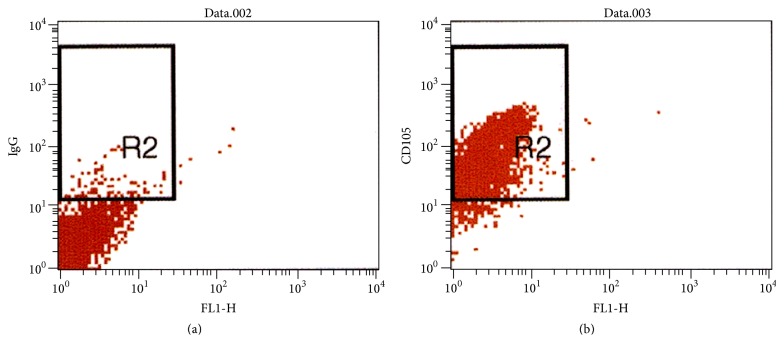
Cytometric analysis of cells that were positively isolated from a sample of bone marrow aspirate obtained from a 34-year-old male nonsmoker. Cells were isolated using the magnetic activated cell sorter (MACS, Miltenyi Biotec) and anti-CD105 monoclonal antibodies conjugated to a metallic residue. Cells were then stained with IgG control antibody or fluorescent monoclonal anti-CD105 antibody. Left panel shows IgG control (gated cells, 2.84%); right panel shows CD105^+^ cells (gated cells, 92.32%). Representative analysis for one out of 24 bone marrow samples.

Cytometric analysis was performed for cells that were positively selected from all samples of bone marrow aspirate. 70–92% of cells were CD105^+^, indicating the efficacy of the isolation method. A representative cytometric analysis for one sample is shown in Figure 1.

### 3.3. CD105^+^ Progenitor Cell Concentration

The mean concentration of BMPCs in bone marrow samples taken from current smokers (1.31 × 10^5^ ± 1.61 × 10^5^/mL) was significantly lower than the mean concentration in nonsmokers (3.52 × 10^5^ ± 2.45 × 10^5^/mL) (*t* = 3.2, *P* = 0.004, Table 3).

## 4. Discussion

BMPCs, also known as multipotent mesenchymal stromal cells, are known to play a central role in musculoskeletal tissue regeneration. Importantly, bone marrow-derived progenitor cells are also known to mediate neoangiogenesis through the secretion of vascular endothelial growth factor (VEGF). Angiogenesis is essential for tissue regeneration, including bone formation and healing [27]. Progenitor cells from fat, placenta, umbilical cord, and muscle have similar, albeit not identical, potential to exhibit multipotent differential capability and to play an important role in mediating inflammation and healing [28–30]. However, the bone marrow remains an important source of progenitor cells for skeletal system self-renewal and fracture repair; thus, BMPCs are essential players in the regenerative capacity of bone and other mesenchymal tissues [31, 32].

In view of the many deleterious effects of cigarette smoking on tissue healing and well-being, we designed this study to determine the effect of smoking approximately a pack of cigarettes per day on the BMPC cell population in bone marrow. We found a significantly lower concentration of BMPCs in bone marrow aspirate from individuals who were current smokers compared to those who described themselves as nonsmokers (Table 3).

BMPC scarcity, as well as technical difficulties in isolating BMPCs from fresh bone marrow samples [33], has precluded the ability to directly quantify the BMPC population in bone marrow. The traditional method of BMPC isolation is based on their unique property of plastic adherence in culture (negative selection), precluding accurate estimation of the BMPC population. In the current study, rapid BMPC isolation from fresh bone marrow samples was accomplished using magnetic activated cell sorting (MACS) based on their display of the CD105 cell marker (positive selection). We have shown previously that this technique is robust for the isolation of BMPCs from bone marrow aspirates [26] and have used cells isolated by this technique for prevention of fracture delay and nonunion in a randomized clinical trial [34].

Normal physiological function of musculoskeletal tissue depends on constant renewal and turnover of a variety of specialized tissues. This process is mediated and orchestrated by BMPCs [22–24]. BMPCs play a similarly important role in mediating healing of musculoskeletal [22, 31–33, 35, 36] and other injured tissues [24, 37, 38]. While BMPCs were shown to have the ability to differentiate into a wide variety of cell lineages that comprise the mesenchyme, recent reports have also focused on their central role as a part of the trophic and immunomodulatory activities in skeletal tissue through secretion of a diverse range of bioactive molecules that exert control as well as direct cell-cell interactions [22–24, 31–33, 37].

Cigarette smoking has been linked to a wide spectrum of pulmonary and extrapulmonary diseases with systemic consequences (reviewed by Yanbaeva et al. [39]). Several studies have shown that cigarette smoking is adversely related to common musculoskeletal complaints such as low back pain [40–42], degenerative disc disease [43–45], cartilage loss and knee pain in patients with osteoarthritis [46], and bone mineral loss and risk of hip fracture [47]. Smoking is also associated with delayed healing of injured skeletal tissues. A review of the literature shows widespread evidence that smoking has a significant effect on fracture repair and union, including bone regeneration, osteointegration, the time required for fracture repair, the risk of nonunion, and the risk of osteomyelitis [1–3]. In addition, smoking is suggested as a risk factor for early failure in hip arthroplasty [48] and dental implants [49].

We found that smoking at least a pack of cigarettes per day is associated with a decreased population of BMPCs in the marrow. We hypothesize that the smaller reservoir of BMPCs in bone marrow contributes to the decreased regenerative capacity of cigarette smokers, which manifests in this wide range of clinical observations.

The major limitation of the current study is the lack of findings that establish a direct mechanism by which cigarette smoking leads to a reduction in the BMPC population in bone marrow and reduced regenerative capacity in the musculoskeletal system. While the effects of cigarette use are clinically documented and possible mechanisms for these effects have been described, further studies are warranted to more clearly establish causation. A minor limitation is our inability to determine any dose relationship between the level of cigarette consumption and the effect on BMPC concentration in this relatively small study population.

While exposure to cigarette smoke carries known as well as unknown effects, we have shown in this study that the concentration of BMPCs in bone marrow, a critical reserve for tissue regeneration and healing, is reduced in individuals who are current smokers.

In conclusion, we hereby show that cigarette smoking is linked to a significant decrease in bone marrow concentration of mesenchymal stem cells. This reduced cell population may contribute to the reduced regenerative capacity of smokers, with potentially important implications for physiological maintenance and repair in the musculoskeletal system.

## Figures and Tables

**Table 1:** Demographic data.

	Smokers	Nonsmokers	*P* value^*∗*^
Mean age (SD)	39 (12.91)	36 (11.87)	0.75
Gender (M : F)	7 : 6	9 : 4	0.42

^*∗*^Student's *t*-test.

**Table 2:** Hematology.

	Smokers (SD)	Nonsmokers (SD)	*P* value^*∗*^
HGB	13.33 (2.07)	12.95 (1.94)	0.63
RBC	4.83 (0.94)	4.45 (0.62)	0.23
MCV	85.43 (7.24)	86.10 (4.35)	0.78
WBC	8.22 (2.48)	8.90 (2.46)	0.49
MONO %	7.49 (2.87)	6.17 (1.72)	0.17
PLT	231.6 (78.4)	313.5 (150)	0.09
MPV	9.27 (1.21)	8.41 (1.42)	0.10

Peripheral venous blood counts from samples collected one day prior to surgery. Mean and standard deviation (SD) are shown.

HGB: hemoglobin; RBC: red blood cells; MCV: mean corpuscle volume; WBC: white blood cells; MONO %: percentage of monocytes in WBC; PLT: platelets; MPV: mean platelet volume.

^*∗*^Student's *t*-test.

**Table 3:** Bone marrow progenitor cell (BMPC) concentration.

	Smokers	Nonsmokers	*t*	*P* value
BMPC concentration (SD)	1.31 × 10^5^/mL (1.61 × 10^5^/mL)	3.52 × 10^5^/mL (2.45 × 10^5^/mL)	3.2	0.004

In each bone marrow sample that was processed, the total number of cells that were recovered was counted. The concentration of BMPCs in 1 mL was calculated by dividing the total cell number by the volume of the relevant sample (in mL). The average BMPC concentration in each study group is presented with the standard deviation (SD).
